# Effect of the hybridization of social and personal responsibility model and sport education model on physical fitness status and physical activity practice

**DOI:** 10.3389/fpsyg.2023.1273513

**Published:** 2023-10-17

**Authors:** Antonio Luis Quiñonero-Martínez, María Isabel Cifo-Izquierdo, Bernardino Javier Sánchez-Alcaraz Martínez, Alberto Gómez-Mármol

**Affiliations:** ^1^Faculty of Sports Science, University of Murcia, Murcia, Spain; ^2^Faculty of Education, University of Granada, Granada, Spain; ^3^Faculty of Education, University of Murcia, Murcia, Spain

**Keywords:** health, secondary education, teaching methods, sedentary habits, teenagers

## Abstract

Physical activity patterns, sedentary habits and obesity levels among children and teenagers are indicators of a worrying reality which has been aggravated by the COVID-19 pandemic. In this context, this study has analysed the impact that new methodologies in Physical Education have on physical health aspects. Two teaching methods, namely, Teaching Personal and Social Responsibility and Sport Education Model, were hybridized, in a Secondary School in Murcia (Spain). Controlled quasi-experimental research was completed with a sample of 76 Secondary Education students aged 12–14 (male: 32; female: 44), developing a hybridization of both models in the experimental group. The *Physician-based Assessment and Counselling for Exercise* (PACE) questionnaire and Eurofit and Alpha Fitness motor tests were run to collect the results. Those showed significant improvement in standing long jump and speed-agility results in the control group. In the experimental group, on the contrary, no significant improvement was registered for either test, but out-of-school physical activity rates were higher. Following this research, it is concluded that both models have positive influence on physical activity habits, but the teaching time devoted to the subject of Physical Education is not enough to improve them.

## 1. Introduction

Since the end of XX century, we have witnessed a struggle to incorporate health education in the Education field (Hurtado-García and Terrón-Bañuelos, 2022). As a matter of fact, Act 2/2006 of Education and Act 8/2013 for the Improvement of Education Quality established a specific block of contents which relates physical activity and health (Block IV. “Physical Activity and Health”) inside the subject of Physical Education. Nowadays, the current law, Act 3/2020, which modified Act 2/2006, (hereon referred to as LOMLOE) includes basic knowledge specific to Physical Education which is related to active and healthy lifestyle.

This relationship is not whimsical as childhood obesity is a global epidemic and a very worrying disease (Saliba and Cuschieri, 2021). This alarming situation has been aggravated by the public health crisis induced by COVID-19 (Babu, 2022). In fact, new terms such as “covibesity” have appeared to refer to this situation (Bagherian et al., 2022). The lockdown during 2020 increased sedentary and inactive lifestyle habits which are among the most influential factors on high obesity levels (Blanco et al., 2019). Furthermore, in general terms, those factors are also motivated by aspects such as the impact of technologies and screen-based activities on leisure time (Lozano-Sánchez et al., 2019).

In Spain, obesity in childhood has been increasing during the last decades reaching alarming levels (De Bont et al., 2022). The main cause is the low physical activity levels that young people present but also other aspects as unhealthy eating habits complicate its eradication (Ramos-Pino and Carballeira, 2022). In Murcia, Carpena et al. (2023) affirm that the situation in Murcia is also worrying being necessary to change this dynamic promoting physical and sport activities in secondary schools in this region.

In this context, studies about obesity and sedentary habits have proliferated as they are more and more frequent among children, pre-adolescents and adolescents (Aparicio-Ugarriza et al., 2020; Jiménez-Parra et al., 2022). At the same time, it can be argued that Physical Education is a powerful resource to change this tendency. Currently in Spain, there are different programs, initiatives and studies (Lozano-Sánchez et al., 2019; Aparicio-Ugarriza et al., 2020) which have similar objectives: to motivate students to change lifestyle habits in curricular and extracurricular time. Some instances are “Active Breaks” which defends the positive relation between short periods of physical activity and concentration (Jiménez-Parra et al., 2022) and “Active Playgrounds” which tries to increase physical activity levels during break time at school, motivating students by creating spaces to play different games accessible for everybody, reducing the star role which balls have traditionally had (Salas-Sánchez and Vidal-Conti, 2020) Also in Spain, but with an international influence, it would be important to mention “Pasos Study” which deals with physical activity, sedentary lifestyle and obesity among Spanish youth, being one of the main goals to improve and increase physical activity levels among children and adolescents (Gómez et al., 2020; Wärnberg et al., 2021).

Besides that, as it can be read in the current Spanish education law (LOMLOE), physical activity has a main role in the subject of Physical Education, being one of the most important objectives to change students’ inactive habits and developing a positive attitude to physical activity. To achieve this aim, it is necessary to consider the recommendation of World Health Organization (hereon referred to as WHO) in terms of physical activity. WHO defends that young people (5–17 years old) should practice 60 min of moderate-vigorous activity every day and spend, at least, 3 days per week doing 60 min of vigorous physical activity (World Health Organization, 2020).

Physical Education, however, was traditionally related to the quantitative development of physical capacities and sport skills and forgetting students’ motivation (Burgueño et al., 2017). This factor added to the presence of technologies in children’s lifestyle has caused students to reject sport practice and physical activity not only during Physical Education lessons but also in their leisure time (Hinojo Lucena et al., 2020). In view of this situation, the above-mentioned approach to Physical Education has proved ineffective, and it has been deemed necessary to work on new methods and strategies to increase the participation, motivation and involvement of students in an active way on Physical Education classes (Burgueño et al., 2017; Hinojo Lucena et al., 2020).

Sport Education Model (hereon, SEM) and Teaching Personal and Social Responsibility Model (hereon, TPSR) are two examples of this changing process (Bessa et al., 2019; Sánchez-Alcaraz et al., 2019). The first one, SEM, is focused on values related to “fair play” throughout sports. In this sense, the main goal is to offer an educational approach to sport, removing the excessive competitive spirit which characterizes institutional sport (Siedentop et al., 2004). Moreover, this model transfers the responsibility from teachers to students allowing them to manage the teaching–learning process development. Furthermore, SEM promotes some feelings and experiences such as the sense of belonging to the group or group cohesion (Calderón-Luquín et al., 2011), motivation and enthusiasm among teachers and students (Calderón-Luquin et al., 2010; Evangelio et al., 2018; Bessa et al., 2019) which are beneficial to students’ holistic development. To meet those objectives, this model applies specific resources and strategies (Fernández-Rio and Menéndez-Santurio, 2017):

▪ To divide students into groups.
▪ To organize the syllabus design in “seasons” which last between 18–20 sessions.
▪ To create different competitions during each season.
▪ To assign roles to each member of the group related to the sport (referee, captain, doctor, …).
▪ To celebrate a final festivity in the last session with a ludic approach.

On the other hand, TPSR is based on social and personal values development through Physical Education (Hellison, 2011) promoting social and personal responsibility, both in Physical Education classes and in their daily life (Escartí et al., 2010a,b; Caballero-Blanco et al., 2013). This model evolves through a sequence of values which ranges from total irresponsibility towards the transfer of values in non-formal contexts (Hellison, 2011):

▪ Level 0: Irresponsibility
▪ Level I: Respect and self-control
▪ Level II: Participation and effort
▪ Level III: Self-direction
▪ Level IV: Leadership and caring
▪ Level V: Transfer

Throughout each level, TPSR develops social values (respect, teamwork and cooperation) and personal values (effort and personal autonomy). The sequence is developed through the sessions being aware that the objectives of each level should be achieved before moving on to the next level (Hellison, 2011; Sánchez-Alcaraz et al., 2019). In the subsection Methods, a deeper description of each element is provided.

A series of factors determined the choice of these two models for this study. It is undeniable that both methods offer high educational value as they contribute to the holistic education of students, overcoming the traditional conception of Physical Education as a field to develop just physical capacities and skills. TPSR and SEM have a potential to transfer values to students, especially responsibility and motivation to students in physical activity and sport context from the Physical Education class to their daily lives (Fernández-Rio and Menéndez-Santurio, 2017). Nevertheless, that values are not developed by themselves in TPSR or SEM; it is necessary to guide the teaching–learning process, using methods, resources and strategies in line with those teaching models (Sánchez-Alcaraz et al., 2020). Both models have similar characteristics which can be combined to get the maximum of their possibilities (Fernández-Rio and Menéndez-Santurio, 2017; Bessa et al., 2019; Opstoel et al., 2020; Rodríguez et al., 2021; Sánchez-Alcaraz et al., 2021) as it will be explained in Method section.

In addition, both models approach the teaching–learning process based on motivational parameters which try to encourage students to do sport or physical activity outside school through positive experience in Physical Education classes (Escartí et al., 2010b; Fernández-Rio and Menéndez-Santurio, 2017). For this purpose, these models place students at the centre of the teaching–learning process, increasing their capabilities for building their own learning and for decision-making, thus improving their autonomy, motivation and participation in Physical Education classes (Opstoel et al., 2020).

Last but not least, this hybridization is common in the Education field as the combination of their characteristics is profitable for both models, being most of them complementary (Fernández-Rio and Menéndez-Santurio, 2017). Understanding hybridisation as the combination or fusion of different teaching–learning models, the characteristics of each one must be considered in a mixed way. In this sense, the methodology to be used should not be based on a single model but on a hybridisation, since the implementation of a single model does not allow to meet all the needs of the teaching–learning process (such as the content or the educational context) but the combination of different models allows to reduce this disadvantage (Evangelio et al., 2017).

There are several examples that have studied the application of both models and its influence on physical fitness levels and physical activity practice. One of them is the research developed by Sánchez-Alcaraz et al. (2021) in a context like this study, in which authors analysed the relation between the personal and social responsibility and physical activity levels on primary and secondary students at a high school in Murcia. In a similar way, also in Murcia, Gómez-Mármol et al. (2017) used a questionnaire to analyse the same relation. In Spain, there are other research as Fernández-Hernández et al. (2021) or Delgado-Floody et al. (2020) studies in which authors related physical activity levels in primary and secondary students with personal and social responsibility and other variables such as motivation. In all of those studies, results showed a positive influence on active and healthy habits.

Nevertheless, it is said that those models, especially TPSR, decrease motor engagement time as they use a vast amount of time in passive actions (meetings, organization, etc.) (Sánchez-Alcaraz et al., 2019; Salas-Sánchez and Vidal-Conti, 2020). For this same reason, short recent studies demand new proposals that hybridize teaching models which add motivational factors to physical activity and at the same time increase physical activity levels (Salas-Sánchez and Vidal-Conti, 2020).

There are also numerous studies that support the relationship between SEM and the increase in physical activity levels thanks to aspects such as improving motivation and predisposition towards the practice of physical activity (Valero-Valenzuela et al., 2020a). For example, Wallhead et al. (2014) analysed the influence of this model in relation to the motivation towards physical practice in the students’ free time. After the application of the SEM, results showed greater motivation and greater predisposition towards the practice of physical activity in non-formal environments. However, these authors stated that, despite this greater provision, the application of the model did not guarantee an increase in physical activity during the leisure, with an appropriate extracurricular outlet being necessary. Along these same lines, Perlman (2010; 2012) also states that the application of SEM has a positive influence on the student motivation. In his study, in which he compared the application of a traditional methodology versus SEM, the author affirm that SEM favors the commitment of the student towards physical practice.

However, there are few publications which attend to physical activity levels or physical fitness status using the hybridization despite the positive results that this combination present (Hastie and Buchanan, 2000). In Spain, Lorente and Joven (2011) developed a research during 12 years proving a positive relation between both models and physical activity practice in extracurricular contexts. More recently, there are more examples that use the hybridization of SEM or TPSR with other levels such as Teaching for Understanding Games model (García-Castejón et al., 2021) or gamification (Melero-Canas et al., 2021).

Considering this context, the aim of this study was to analyse the effect that the hybridization of TPSR and SEM have on secondary education students’ physical fitness levels and physical activity practice. The characteristics of both models were considered and hybridized, Eurofit and Alpha Fitness motor tests were implemented to assess physical fitness levels and PACE questionnaire was applied to assess physical activity practice, not only within the educational context but also out of it.

## 2. Materials and methods

### 2.1. Study design

Semi-experimental controlled research (own research) was carried out in a High School Centre in Algezares (Murcia, Spain) (Thomas et al., 2011). This school was selected by convenience, as the teacher leading the educational intervention was familiar with TPSR and SEM. The early-stage design was produced in cooperation with him to establish the working program because it had to adjust to the syllabus design for the subject. After he agreed, the head teacher and the principal of the high school gave their authorization. Legal tutors and students were duly informed about this intervention, and they also agreed to participate, by means of legal documents proposed by the Ethics Committee of University of Murcia (ID: 4117/2022). Furthermore, this Committee assessed the study design positively and allowed this pedagogical intervention.

### 2.2. Sample

This study was developed with 4 groups of second grade of Secondary School. At first, a group of 107 students were selected, but only 76 could fulfil the complete process. Results from students were deemed non-valid if (a) students had not complete pretest and/or post-test (b) students were absent from more than 3 sessions and/or (c) students did not do a single test correctly – whether theoretical or practical.

Those 76 students, aged 12 to 14, (male: *n* = 32; female: *n* = 44) were randomly divided into a control group (*n* = 36) and an experimental group (*n* = 40) formed by class-groups. The former followed a traditional methodology while the latter put into practice the hybridization of TPSR and SEM. All of them had two Physical Education classes (60 min each) per week, using the same sports facilities (school indoor gym and an outdoor multisport court).

### 2.3. Instruments

The following instruments were used for data collection. These instruments have been widely used in scientific literature due to their high values of validity and reliability, low cost and easy application.

#### 2.3.1. Physical activity

This variable was assessed through the questionnaire *Physician-based Assessment and Counseling for Exercise* (PACE) (Kolimechkov, 2017). This survey has two questions: the first one refers to the level of activity that a student normally does, and the second one aims at the specific physical activity level in a punctual sense (the past 7 days). Each question has only one possible answer (0–7) which represent the number of days per week. The questionnaire was completed in class just before and after the experiment, with the teacher present in the classroom. Students were allowed to ask questions after the explanation was concluded and there was no time limit. The results were kept anonymous.

#### 2.3.2. Physical fitness

To analyse physical fitness levels, it was necessary to refer to samples from Eurofit and Alpha-Fitness battery of tests. Standing long jump, speed-agility and cardiorespiratory endurance tests were selected as the teacher had previously included those motor tests in his syllabus design so we used them not to interfere in the normal development of the subject. Eurofit standing long jump test together with Alpha Fitness cardiorespiratory endurance and speed-agility test were the ones selected to assess the students’ physical fitness levels. All of them were applied the week before and the week after the intervention process both in control and experimental groups. Each test had some specific instructions:

1. Standing long jump test (Castro-Piñero et al., 2010): one by one, each students put their feet together behind a line marked by the teacher. When they were ready, they jumped as far as possible keeping their balance. After that, the distance was measured from the line to the student’s heels.
2. Speed-agility test (Ortega et al., 2008): before the practice, a circuit was prepared using two cones and demarcating a 10 m distance between each one. The students had to begin behind the first cone. The teacher started a countdown, saying “three, two, one, go.” When students heard “go” they started running as fast as possible touching each cone. They had to run to the second cone and back, five times in all.
3. Cardiorespiratory endurance (Liu et al., 1992): all the students started behind a line marked by the teacher. Before starting, the teacher explained that they were going to follow a recorded track which would give them instructions. Moreover, the teacher explained that they would have to run from the starting line to another line (located 20 m away) as many times as they could. They had to follow the recording which would indicate the moment when they had to stop and restart the run. If they reached the line after they heard the sound, the test was over for them.

Each motor test was explained beforehand in three different ways:

a) An oral explanation in class, emphasizing the key points and rules of each test.
b) Videos which allowed students to see how to act in each test.
c) A new oral explanation just before the motor test took place.

Along with the explanation of the tests, it was important to make students aware that it was not a competition, making emphasis on the importance of accuracy during their practice.

Before each test, the teacher led a warm-up process orientated to the specific motor test. After that, students had the opportunity to do each test twice, keeping the maximum mark.

### 2.4. Procedure

For 3 months (from January to March) and 17 sessions, the experimental group developed the hybridization of TPSR and SEM integrated into the school syllabus design. For that reason, sessions were organized in three teaching units which were based on three alternative games and sports:

▪ Teaching unit 1: based on Colpbol sport; 6 sessions. In this unit the aim was to develop respect and participating and effort which correspond to TPSR level 1 – spanning 4 sessions – and TPSR level 2–2 sessions. Those overlapped the first phase of SEM (presentation and introduction of model characteristics: roles, competition…) and also the second phase, namely preseason, which was based on practice directed by the teacher.
▪ Teaching unit 2: based on racket games; 6 sessions. It focused on TPSR level 2–2 sessions – and TPSR level 3, which dealt with personal autonomy – 4 sessions. This level concerned the autonomy of students, but the teacher made emphasis on respectful behaviours that had been worked on during the previous teaching unit. As for SEM, the second phase was completed, and third phase (*season*), based on competition, was developed.
▪ Teaching unit 3: based on Frisbee Ultimate sport; 7 sessions. The last teaching unit focused on TPSR level 4 – helping others – 4 sessions. The rest of the unit was programmed to reinforce previous TPSR levels. Regarding SEM, phase three was over and a final festivity was prepared and celebrated in the last session (phase 4).

On the other hand, the control group followed the same syllabus design, based on the same games and sports for the same period of time but putting into practice the traditional methodology proposed by the teacher. This methodology always observed the same session structure divided into three parts (Seners, 2001):

▪ First part (10–15 min): students went to the meeting point (locker room) where attendance was checked. After that, a warm-up routine was followed by students.
▪ Second part (30–35 min): at least three games or activities based on the arranged sport were played involving technique and tactic elements.
▪ Third part: (10–15 min): students developed a calm-down routine before returning to their reference classroom.

A unique teacher directed the teaching–learning process for both groups (control and experimental). The two syllabus designs were based on blocks I, II, and IV (Table 1) which were related to the following curricular elements (Ley Orgánica, 2013), (Ley Orgánica 8/2013, de 9 de diciembre, para la mejora de la calidad educative (LOMCE), 2013):

**Table 1:** Curricular framework (Decreto, 2015).

Block of contents	Content	Key competence	Stage objective
Block I. Physical fitness and health	Physical capacities development	Social competence	K
		Scientific competence	H
Block II. Games and sports	Knowledge of technique, tactic and rules of sports	Social competence	A, C
		Learning to learn	K
Block IV. Common elements	Rules acceptance and elaboration	Social competence	A, C
	Team-work and active participation	Social and learning to learn competences	B

#### 2.4.1. Model hybridization

The process of hybridizing TPSR and SEM had to comply with specific curricular elements. The characteristics that were considered during the process will be outlined now so that the merge can be fully understood.

Regarding TPSR, there were two standing characteristics: values progression and session model (Hellison, 2011; Sánchez-Alcaraz et al., 2016; Fernández-Rio and Menéndez-Santurio, 2017).

▪ *Values* progression: the main objective of this model is to gradually develop personal and social values throughout systematic progression throughout the whole intervention process (Hellison, 2011). This progression includes 5 levels which correspond to respect (level 1), participation and effort (level 2), personal autonomy (level 3), helping others (level 4) and the last level which consists in taking responsibility out of the Physical Education class context (level 5).
▪ *Session structure*: every session was divided into four parts as it is established in this model (Menéndez and Fernández-Rio, 2017).
o The first part is related to the awareness process of each value which is worked on at that moment. This part normally lasts from 10 to 15 min, including the time for getting to the outdoor court or the gym and checking attendance, among other class routines. During this time, the teacher introduces the session aims related to values, using an informative poster, displayed permanently on the gym wall.
o The second part, “activity plan,” is based on responsibility in action which means that students should put into practice the behaviours and attitudes explained by the teacher in part one. Therefore, for 25 to 35 min motor games and modified games are suggested, allowing students to learn the technique and tactic of each sport, as they ae given more and more responsibility for their own preparation using SEM roles together with autonomy practice, also typically of this second teaching model.
o The third part (from 10 to 15 min) consists of a meeting in which students discuss the most important elements and events which have taken place during the session. Students with referee roles direct an assembly sanctioning the behaviours which were against TPSR values and reinforcing the positive attitudes of their classmates.
o The last part is related with session assessment. For 2 min students individually draw conclusions from the previous part and assess the session using thumb test.

All these parts were organized into 60 min classes, including some time to go from the classroom to the court, to collect the material or to allow for students’ personal hygiene. In addition, this fix structure improved responsibility-level results among students as they better acquire the routines thus feeling confident and motivated (Prochaska et al., 2003).

The second model in the tandem, SEM, is based on the sport teaching process focused on providing gratifying experience to students and understanding the concept of fair play as a key point (Carriedo et al., 2022). To achieve its goals, this model puts into practice different strategies and dynamics (Siedentop et al., 2004; Fernández-Rio and Menéndez-Santurio, 2017):

The teaching units are called “seasons” which have a longer duration than traditional units. This study comprised the development of only one season overlapping three teaching units (one per sport/game).

▪ Students are divided into stable teams. Each team should have the same number of students. In this case, it was necessary to form three slightly different groups per class (5–6 students).
▪ Each member of the group has a responsibility role which changes every three sessions. For this study, roles were *referee, assistant referee, captain, journalist, coach and assistant coach*. Each role has specific functions which must be displayed at all times, easily visible on the wall of the class and indoor gym (for instance, captain had to collect the material; coach and assistant coach had to receive instructions from teacher and explain them to their teammates; journalist had to write one 300-words article after each session, referees and assistance referees must control match development taking decisions considering rules …).
▪ Sports and games are modified to adapt motor situations to the possibilities and limitations of students. Some strategies to achieve that in this context were to adapt spaces, material or time or rules (for instance, when the experimental group was playing racket games, the ball could touch the ground more than once). Throughout these modified situations, technique and tactics are included as part of the teaching–learning process.
▪ Different competitions are developed during each season. Nevertheless, this model prescribes different scoring taking into account not only the conventional point system related to each sport, but also including aspects related to the values which have been introduced previously.
▪ At the end of the season, a final festivity takes place. Each team competes in a ludic atmosphere, celebrating the end of the season. Students were allowed to bring banners, to design equipment or to create a choreography to introduce the team before the competition.

## 3. Results

### 3.1. Physical activity

Table 2 shows the results obtained both in control and experimental groups concerning the number of days that students do physical activity in a normal week. In this sense, pretest results showed that 12.5% of control-group students did not do any exercise during a normal week, being 15% the students who did not do any exercise in the experimental group. In contrast, post-test results showed that 3 control-group students (7.5%) did not do physical activity in a normal a week while 2 (5%) experimental-group students chose this option, post-test results being lower than pretest results in both groups.

**Table 2:** Question 1. Physical activity level in a normal week (control and experimental group).

Days per week	Pretest		Post-test	
	*N* (%)		*N* (%)	
	Control	Experimental	Control	Experimental
0	5 (12.5)	6 (15)	3 (7.5)	2 (5)
1	4 (10)	3 (7.5)	2 (5)	2 (5)
2	4 (10)	6 (15)	7 (17.5)	7 (17.5)
3	6 (15)	4 (10)	12 (30)	9 (22.5)
4	9 (22.5)	6 (15)	3 (7.5)	7 (17.5)
5	6 (15)	4 (10)	7 (17.5)	4 (10)
6	5 (12.5)	6 (15)	3 (7.5)	2 (5)
7	1 (2.5)	1 (2.5)	3 (7.5)	3 (7.5)

In the control group the highest percentage in pretest was associated with option “4 days per week” with a total of 9 answers (22.5%), while in the experimental group the maximum number of answers were divided into zero, two, four and 6 days per week with a total of six answer (15%) in each field. As regards post-test, in both groups, the highest percentage was associated with the answer “3 days per week” as 12 (30%) students selected this option in the control group and 9 (22.5) did in the experimental group. For both, this option obtained the highest mark attending to pretest and post-test results. There were no significant differences between control group and experimental group neither in pretest stage (*p* = 0.748) nor in post-test stage (*p* = 0.996).

Table 3 shows results referred to question 2, related to the number of days that the students had done physical activity in the previous week. Pretest results showed that 7 students (17.5%) from the control group said that they had not done any exercise during the previous week, this result being higher than the same option for Question 1. However, 3 students (7.5%) said that they had done physical activity every day in the previous week while in Question 1 only one student selected this option. Regarding the experimental group, it can be observed that 9 students chose option “4 days per week” in pretest while 11 students selected that option in post-test. The same table shows that in post-test the option “0 days per week” was selected by 1 student (2.5%), from the control group, this being the minimum percentage register. There were no significant differences between control group and experimental group neither in pretest stage (*p* = 0.305) nor in post-test stage (*p* = 0.727).

**Table 3:** Question 2. Physical activity level in the previous week (control and experimental group).

Days per week	Pretest		Post-test	
	*N* (%)		*N* (%)	
	Control	Experimental	Control	Experimental
0	7 (17.5)	5 (12.5)	1 (2.5)	2 (5)
1	3 (7.5)	1 (2.5)	2 (5)	0 (0)
2	7 (17.5)	5 (12.5)	9 (22.5)	6 (15)
3	8 (20)	3 (7.5)	6 (15)	8 (20)
4	2 (5)	9 (22.5)	7 (17.5)	11 (27.5)
5	6(15)	7 (17.5)	5 (12.5)	5 (12.5)
6	4 (10)	4 (10)	6 (15)	1 (2.5)
7	3 (7.5)	2 (5)	4 (10)	3 (7.5)

Table 4 shows that physical activity levels increased in the control group and experimental group if the results in pretest and post-test are contrasted. As it can be seen, the mean was superior in both questionnaires and in both groups. In fact, the differences between the results of the second questionnaire for the control group were significative (*p* = 0.02), this being a medium effect size (0.4) as the control group was formed by less than 50 people (Escartí et al., 2013).

**Table 4:** Physical activity levels.

Group	Number of days	Pretest (M ± SD)	Postest (M ± SD)	*Z*	*P*	*d*
Control	Normal week	3.32 ± 1.99	3.45 ± 1.89	−0.476	0.634	0.1
	Last week	3.10 ± 2.22	3.88 ± 1.90	−2.330	0.020	0.4
Experimental	Normal week	3.17 ± 2.16	3.58 ± 2.05	−0.756	0.450	0.2
	Last week	3.44 ± 1.81	3.67 ± 1.66	−0.077	0.938	0.1

However, data in Table 4 reveal that the experimental group did not experience any significant change comparing pretest and post-test results. Even though there were higher results regarding the number of days that students did physical activity, the effect size was small for Question 1 (0.2) and Question 2 (0.1).

### 3.2. Physical fitness

Table 5 displays results for control group and experimental group students in motor tests (speed-agility, standing long jump and cardiorespiratory endurance). Comparing both groups, it shows that the experimental group achieved superior pretest results except for speed-agility test. The control group started from lower pretest results but experienced an improvement in all categories results after the intervention. The experimental group obtained higher results in speed-agility (M = 8.80, SD = 1.14) and cardiorespiratory endurance post-test (M = 2210.38; SD = 487.76) but had a decrease in standing long jump test as it can be seen in Table 5 (M = 1.58; SD = 0.27).

**Table 5:** Differences between control and experimental group (pretest and post-test).

Test	Group	Control (M ± SD)	Experimental (M ± SD)	*Z*	*p*	*d*
Speed-agility	Pretest	9.16 ± 1.08	8.5 ± 0.76	−2.073	0.038	0.7
	Postest	9.38 ± 1.07	8.8 ± 1.14	−0.152	0.879	0.53
Standing long jump	Pretest	1.40 ± 0.25	1.65 ± 0.25	−2.508	0.012	1
	Postest	1.46 ± 0.25	1.58 ± 0.27	−1.360	0.174	0.46
Cardiorespiratory endurance	Pretest	1758.8 ± 368.76	2187.5 ± 418.4	−0.530	0.596	1
	Postest	1970.3 ± 1314.61	2210.38 ± 487.8	0.166	0.868	0.2

Effect size (Cohen’s D).

Table 5 also shows that differences between the first and the second test were significant for the control group in the standing long jump test (*p* = 0.01) and the speed-agility test (*p* = 0.04). The same tests show non-significant differences in the experimental group in both categories (*p* = 0.17 and *p* = 0.88). Likewise, the cardiorespiratory endurance test proved no significant difference, neither in the control group (*p* = 0.6) nor in the experimental group (*p* = 0.87). Focusing on those differences, it is shown that a large effect size (*d* = 1) was obtained from calculating the average of control and experimental standing long jump and cardiorespiratory endurance pretest results. In the same way, speed-agility test showed a large effect size (*d* = 0.7) between control and experimental pretest results. On the other hand, post-test results did not show those differences, the largest effect size being the ones registered in speed-agility (*d* = 0.53) and standing long jump tests (*d* = 0.46), which represents a medium effect size. Finally, cardiorespiratory endurance post-test results showed a small effect size between the averages of both groups (*d* = 0.2).

## 4. Discussion

The main objective of this study was to analyse the influence that the hybridization of TPSR and SEM has on students’ physical activity levels and physical fitness status. With that purpose, it was decided to analyse habitual and punctual physical activity using PACE questionnaire.

Regarding physical activity levels, it could be said that there was significant improvement in the control group activity levels (habitual and punctual) but not in the experimental group. Nevertheless, this second group also showed an increase on both results.

Those results go in line with previous research which showed non-significant results for the experimental group in this dimension after the application of SEM (Pérez-Pueyo et al., 2021). Despite the results obtained with PACE questionnaire, these authors affirm that physical activity levels improved applying SEM, as this model has a positive influence on other variables such as participation or sportsmanship. Perlman (2011) also affirm that the application of SEM has a positive influence in students’ motivation. In the case of the application of TPRS, Gómez-Mármol et al. (2017) observe a relationship between increased accountability and improved levels of practice. Furthermore, in the work of Gómez-Buendía et al. (2022) they also conclude that both models (TPSR and SEM) and their hybridisation favour student enjoyment. Therefore, it can be considered that these variables encourage students’ willingness to participate in physical activities and sports.

As well as this previous study, other research also defends the relation between the improvement of those variables and physical activity levels (Valero-Valenzuela et al., 2020b). In this study, which deals with different active models (SEM and gamification), authors justified this relation mentioning that the improvement of students’ self-confidence and motivation rendered them more predisposed to participate in physical activities, not only in school but also in non-formal contexts. Pritchard et al. (2015) also affirm that a season of SEM has a positive impact on physical activity levels in non-formal contexts as their quantitative results showed better physical practice levels in students’ spare time such as summer.

After Valero-Valenzuela et al. (2020b) and Kurt et al. (2017) interventions, significant improvements were obtained in terms of physical activity levels, which contrast with those of our study. Indeed, their study was different to ours, as they applied the hybridized model oriented directly to developing physical fitness using specific techniques for that purpose, whereas our current study used the hybridization developing different alternative sports to achieve that goal.

Considering another study, which analyse the influence of TPSR on physical activity levels, the improvement on dimensions such as personal and social responsibility proved to have an impact on students’ perception of physical activity (Sánchez-Alcaraz et al., 2021). However, physical activity levels results were medium-low and there were no significant changes in any dimensions (punctual or habitual). In this sense, Hastie and Buchanan (2000) explain that the relation between this model and physical activity levels was indirect as the results and improvement were not only derived from the intervention itself, but also from the experiences or feelings such as enjoyment, task-involving and self-perception.

Consequently, it is important to emphasize that the results shown for both groups in our study allow us to understand that most of the students did physical activity 3 days per week, while the percentage of students who did exercise every day of the week as WHO recommends (World Health Organization, 2020) attained the minimum rate. In the light of that information, this study supports the current educational guidelines stating that all teachers should be able to encourage students to do sports and physical activity in their spare time. In fact, despite Physical Education being the specific area which deals with physical activity and health, this should be considered as transversal curricular content which should be included in each subject (Ley Orgánica, 2020), (Ley Orgánica 3/2020, de 29 de diciembre, por la que se Modifica la Ley Orgánica 2/2006, de 3 de Mayo, de Educación (LOMLOE), 2020).

Regarding physical fitness status, motor tests were run before and after intervention in both groups. On one hand, the control group produced better results after intervention, the difference being significant on standing long jump and speed-agility test. On the other hand, there were no significant improvements in the experimental group, which even showed a decrease in standing long jump test.

Those results might clash with a study based on physical activity (Pérez-Pueyo et al., 2021) which established a clear relation between the self-concept and self-confidence that an individual has and their physical activity level and physical fitness status. In addition, Carriedo et al. (2022) showed a direct relation between each dimension and the motor test results. They argue that motor tests in the educational field are influenced by personal self-confidence, and that is one of the elements which is worked on through the implementation of TPSR and SEM.

In fact, other research proved that SEM has a positive influence on the development of physical fitness throughout motor tests (Farias et al., 2018). Their authors defend that this kind of methodologies increase the motivational level and the interest in those motor tests. Moreover, Kurt et al. (2017) claim that this model allows students to discover their own possibilities and limitations increasing their confidence. However, this study did not actually test the quantitative results of motor tests.

Interestingly, better results in control group when applying physical fitness motor tests were also found in previous investigations (Rosa et al., 2019; Carriedo et al., 2022). There the control group students got better quantitative results in standing long jump, speed-agility and cardiorespiratory endurance tests as it happens in our study. The experimental group, however, showed a decreased in standing long jump test and non-significant improvements on cardiorespiratory endurance and speed-agility tests, similarly to the outcome reflected in our study.

The consistency of our results with the findings of Rosa et al. (2019) led us to reconsider and relativize our first hypothesis to explain them. Initially, the non-significant results in the experimental group were attributed to the public health crisis caused by COVID-19 pandemic. In Spain, during 2021, people who were infected by this virus had to stay at home for at least 10 days, as regulated by health authorities. This restriction meant that some students could not finish the study or could not complete all the sessions which were programmed. Other constrictions such as not sharing the material or not being able to touch each other also limited the normal course of the intervention.

However, these results could be pinpointing the fact that both models have some limitations that should be considered. Firstly, TPSR dedicates plenty of time to non-physical activities such as meetings, reflection or self-evaluation in each session. This could be a reason for not having better results in the experimental group as the main aim of this model is not related to the physical dimension of the human being. In the same way, SEM needs a vast amount of time for non-physical elements, such as organizing the competition, creating some material for the final festivity and holding meetings to resolve possible conflicts. This does not mean that the hybridisation of the two models is detrimental to motor development. This hybridisation considers the whole person and therefore focuses not only on the motor domain, but also on all other domains. In this sense, Pan et al. (2019) conclude that the hybridisation of TPSR-SEM increases learning in the motor, cognitive and affective domains. According to the authors, sport self-efficacy, passion for sport, responsibility and game performance improve.

Added to the limitation to the development of physical fitness which are inherent to the models chosen for the hybridization, and the non-inherent health restrictions in force at the time of the intervention, there was another factor which altered the course of the intervention as far as physical fitness development is concerned: unanticipated external agents such as weather. At this time of the year (January, February, and March) the practice of physical activity outdoors was difficult. On rainy days, it was not possible to use the uncovered court of the school where students have enough space to develop their physical skills comfortably, especially in sports such as Colpbol or Ultimate Frisbee. Instead of that, it was necessary to use the multiuse indoor gym which has rather limited space. To adapt to those conditions some alterations to the program were required. In this sense, Casado-Robles et al. (2020) point out that the improvement of physical fitness can depend on many factors, but it can have positive effects if there is adherence to the practice of physical activity. To this end, Segovia and Gutiérrez (2020) recommend programming the SEM model for a longer duration to encourage students to be physically active.

Finally, another aspect that could have influenced the results of our research both in terms of physical activity levels and physical activity status was the lack of time that this intervention had each week. Considering that this study is analysing healthy habits in students’ daily life, and trying to improve them in most cases, 2 hours per week could arguably not be enough to change an unfavourable situation. In the same way, a previous study defends this idea that time devoted to Physical Education in Spanish educational system should be increased (Hastie and Buchanan, 2000; Pérez-Pueyo et al., 2021).

## 5. Conclusion

The effect that the hybridization of SEM and TPSR has on secondary education students’ physical activity routines and fitness levels has been analysed in this study. Eurofit and Alpha Fitness motor test together with PACE questionnaire have been the tools implemented to obtaining the data for the research.

The study was motivated and justified by the current concern about teenagers and children’s health and their growing tendency to choose sedentary activities in their free time – a concern which is acknowledged and reflected in Act 3/2020 (LOMLOE, 2020). The hybridization of those specific models, TPSR and SEM, also responded to our perception of the need to bring the subject of Physical Education closer to up-to-date teaching methods based on the concept of integral or holistic education.

Over the last two decades, new active methodologies have been appearing, trying to put the student at the centre of the teaching–learning process overcoming the traditional conception of Physical Education exclusively focused on physical possibilities and capacities. The new methods take into consideration students’ individual necessities, and shifts focus from physical skills to health as a vital educational issue.

Before this study, SEM and TPSR had been applied in scientific research and there is bibliography in which SEM and TPSR prove effective in combination with the physical dimension of Physical Education to develop mental, social and emotional health. However, there are not so many studies which include those two models as a developmental tool of healthy habits as our research has done.

Considering the purpose of the research, the results of these study have concluded that the physical activity levels increased in the control group and experimental group, also there were significant improvements in standing long jump and speed-agility results in the control group. In the experimental group, on the contrary, no significant improvement was registered for either test, but out-of-school physical activity rates were higher.

However, implementation of PACE questionnaire did not provide complete information about students’ physical activity habits. Most of them said that they did activity at least 3 days per week, but data are not specific as to what kind of physical activity they referred to, differentiating between vigorous and moderate as WHO normally does. In addition, it would have been useful to know the physical activity context so as to learn more about those habits, taking into account, for instance, if students were engaged in out-of-school activities related to sports and exercise, if their practice was self-directed and informal, or if, on the contrary, they only did physical activity in Physical Education classes.

Another key factor was time. After this intervention we can ascertain that 2 h per week of Physical Education classes is not enough time to encourage students to do physical activity out of school and, even less to get them to do the amount of physical activity recommended by WHO. This research thereby supports the necessity to follow the current educational legal framework which reflects this situation establishing physical activity and health as transversal contents. Hence, it is not only Physical Education teachers’ responsibility to encourage healthy habits, but it should also be considered as a collective issue for every agent involved in students’ education and development.

In this sense, our research can shed light to the above-mentioned need in the education field. It is really important to use Physical Education as a source of motivation to encourage students to do physical activity in their free time and integrate that idea in other subjects too as transversal content. Our hybridization of TPSR and SEM obtained its most positive results when measuring the impact of our intervention in terms of physical activity frequency among our student sample, which in fact confirms previous research which have shown that using active methods in class such as SEM could have positive results in terms of motivation and physical activity levels in spare time (Spittle and Byrne, 2009; Merino and Mora, 2022) Thus from this study, it is considered appropriate to encourage teachers to apply those active methods and models to their own classes in order to comply with the current educational requirements and to encourage students to practice physical activity and sport in their spare time, considering physical activity as a means to instill physical, mental and social health. Moreover, by applying this hybridization during break-time through school-level organized competitions, schools would be directly palliating this disconnection between free time and physical activity among some of their students and bringing secondary school education closer to the guidelines recommended by WHO.

This research could be considered a pilot study that would be useful as a referent for further publications. As our study did not produce significant results, it is recommended to keep analysing this hybridization to get more results which relate the effect of TPSR and SEM to physical activity levels while still adhering to healthy habits as the main focus.

Future research might be able to provide deeper insight into the weaknesses of TPSR when applied to raise physical activity levels and physical fitness status so that they can be mitigated. Following bibliography, that mitigation would enhance the standing characteristics of SEM such as autonomy, participation in training and competition, and would allow us to reduce inactive time in Physical Education classes. For further investigations, it is also recommended to analyse the relation between the motivation parameters of SEM and TPSR and the amount of physical activity that students do in their leisure time.

Starting our research based on a syllabus design that was already in use at the school where the intervention took place proved positive for the development of our intervention since it already abided by Spanish educational legislation and did not interfere in the normal course of the subject of Physical Education. However, the shortcomings identified in our intervention, should be useful for upcoming studies to preclude the same difficulties.

Ideally, the intervention should be designed avoiding conditions dependant on external factors, such as the weather and other non-intrinsic restrictions. Moreover, the duration of the intervention should be longer as it has been argued in this research. Additionally, it would be useful to use a valid scale to assess physical fitness in order to quantify more precisely the extent to which results obtained in different moments have increased or decreased.

Investigations which defend a positive relation between physical activity levels and levels of satisfaction and motivation in the subject of Physical Education lessons were a starting point and determined the approach of this research. We conclude it now with the conviction that the whole educational system can play a role in the development of healthy active habits, so that physical activity and healthy habits pervade and transcend the context of formal education.

## Data availability statement

The raw data supporting the conclusions of this article will be made available by the authors, without undue reservation.

## Ethics statement

The studies involving humans were approved by Ethics Committee of University of Murcia (ID: 4117/2022). The studies were conducted in accordance with the local legislation and institutional requirements. Written informed consent for participation in this study was provided by the participants’ legal guardians/next of kin. Written informed consent was obtained from the individual(s) for the publication of any potentially identifiable images or data included in this article.

## Author contributions

AQ-M: Conceptualization, Data curation, Formal analysis, Investigation, Methodology, Project administration, Writing – original draft, Writing – review & editing. MC-I: Conceptualization, Data curation, Formal analysis, Investigation, Methodology, Project administration, Writing – original draft, Writing – review & editing. BS-AM: Conceptualization, Data curation, Formal analysis, Investigation, Methodology, Project administration, Writing – original draft, Writing – review & editing. AG-M: Conceptualization, Data curation, Formal analysis, Investigation, Methodology, Project administration, Writing – original draft, Writing – review & editing.
